# Minimally Invasive Swine Experimental Model for the *In Vivo* Study of Liver Metabolism of Drugs

**Published:** 2012-10-11

**Authors:** O Piazza, R Romano, G Scarpati, C Esposito, E Cavaglià, M Corona

**Affiliations:** Department of Medicine, Salerno University; * AORN Cardarelli, Napoli

**Keywords:** liver, hepatic veins, remifentanil metabolism

## Abstract

**Purpose:**

To develop a clinically relevant porcine model for the study of hepatic metabolism of drugs by means of hepatic vein catheterization.

**Materials and Methods:**

review of literature and elaboration of a hypothesis, design of an experimental method.

**Results:**

recent clinical studies were conducted by withdrawing cirrhotic patients’ blood from right hepatic vein during hepatic vein pressure gradient measurements. Basing on our personal clinical experience and evaluation of research needs, an experimental model is proposed.

**Conclusions:**

contemporary measurement of peripheral and hepatic concentration of drugs by peripheral vein and hepatic vein catheterization can be used to create a reliable and reproducible porcine model to study liver metabolism of drugs in vivo.

## I. INTRODUCTION

Liver is central in drug metabolism in several ways and, as a consequence, hepatic impairment alters drugs availability. Porto-systemic shunting, i.e., decreases the first-pass metabolism of a drug and lead to increased oral bioavailability of highly extracted drugs; impaired production of drug-binding proteins can altered distribution and, furthermore, the activity and capacity of hepatic drug metabolizing enzymes might be affected to various degrees in patients with acute or chronic liver disease. The disposition of a large number of opioid drugs, which are among the most important “weapons” to fight pain in the anaesthesiology and intensive care practice, is modified in the presence of hepatic impairment but the pharmacokinetics of phenylpiperidine opioids such as fentanyl, sufentanil and remifentanil appear to be unaffected in hepatic disease [1].

Since liver is the major organ of drug metabolism, Orthotopic Liver Transplantation (OLT), and in particular its anhepatic phase, provides an unique opportunity to determine if a drug is metabolized in other sites, such as the remifentanil case [2].

Nevertheless, OLT is time-consuming and complex. It has a significant influence on the body internal environment and on the circulation. The characteristics of blood loss, fluid replacement and body temperature during various surgical stages are different and all these factors may have influences on pharmacokinetic parameters. Another technique to study *in vivo* liver metabolism of drugs and in particularly of anaesthetics is highly desirable, consequently.

For these reasons, we propose to study pharmacokinetic of opiods using an experimental model which is based on the withdrawal of blood from the hepatic vein, in normal and pathological conditions, permitting the comparison with the concentration of drugs in peripheral serum and the calculation of the liver extraction ratio.

## II. MATERIALS AND METHODS

Review of literature was conducted searching PubMed database for the terms “hepatic vein catheter and drugs” (37 papers), “HVPG and drugs” (40 papers).

All the matching papers were revised separately by two independent reviewers (OP, RR) who performed trial selection and quality assessment.

Clinical application of the method was tested in two patients who required a deep sedation to perform the hepatic venous pressure gradient (HVPG) measurement and who consented to participate in a clinical research for remifentanil metabolism (data not published) by two experienced physicians (CE, EC) [3].

Evaluation of published experimental methods to measure remifentanil liver metabolism or hepatic extraction of other drugs was conducted searching PubMed database for the terms: “experimental model to study liver metabolism of remifentanil “ (1 paper) “and pig experimental model to study liver metabolism of drugs” (41 papers), “animal experimental model to study liver extraction of drugs” (23 papers). All the matching papers were revised separately by two independent reviewers (OP, GS) who selected the appropriate and focused scientific works.

## III. RESULTS

We included full-text clinical trials in patients with cirrhosis and portal hypertension. The primary outcome was mean change in drug concentration between hepatic venous blood and peripheral blood. From 77 citations, 2 clinical trials [4,5] and 1 experimental paper [6] were included. Data retrieved are showed in table 1.

In two patients, who volunteered to participate a clinical study and who needed HVPG for their clinical condition, peripheral and right hepatic vein blood withdrawals were taken to measure remifentanil serum concentration before and after HVPG without any inconvenience.

As already existing experimental model is concerned, 2 papers [7,8] only fitted partially our criteria: results are described in table 2. Papers in which liver transplant was described or tissues and cells ex vivo were used, were excluded.

From the exposed data, we elaborated the following hypothetical experimental model to realize a minimally invasive, repetitive method to study drug extraction rate in pigs and we named it ETHER (Experimental model To study Hepatic Extraction of Remifentanil).

Pigs are the selected animal for this protocol, because of their availability and the similarity existing between porcine and human liver [9]. Pigs used for this protocol, females with a weight about 60 kg, come from authorized structure (Harlan, Charles River) and are carried with suitable means to the laboratories prepared at the research center about 24 hours before the beginning of the experimental procedures. Then every pig is subjected to a clinical examination to evaluate their health state.

On the first day of the study, blood samples are collected to evaluate liver and renal function. Blood samples are collected from internal jugulair vein after an adequate sedation with tiletamine hydrocloride plus zolazepam 2,5 mg/Kg by an intramuscular injection in the neck region. After 10 minutes pigs are placed in dorsal recumbency to perform blood sampling. After overnight fasting, the study animal is transferred to the experimental OR, where X Rays, angiographic table and complete hemodynamic equipment is available.

Study measurement is done under sedation and monitoring for vital signs (including heart rate, arterial blood pressures, digital oxygen saturation, and ECG). Periodically we monitorize: hemogasanalysis, hematocrit, electrolytes and glycemia.

A possible anaesthesia protocol could by the following:

1. Premedication: in the stall an intramuscular injection in the neck region with tiletamine hydrocloride and zolazepam 2,5 mg/Kg plus atropine 0,025 mg/Kg is performed. After 10 minutes, pigs are clean, taken to the operating room by a special stretcher, and positioned on the operating table.
2. Induction: in the animal in sternal recumbency an incannulation of the marginal ear vein is performed with an adequate angioacath. After an intravenous injection with propofol (6 mg/Kg) plus ketamine hydrochloride (15 mg/kg) plus atropine sulfate (0.04 mg/kg) and the evaluation of palpebral and lingual reflexes, an oro-tracheal intubation is performed. The correct position of the orotracheal tube is confirmed by the auscultation of the right and left lung fields. Pigs positioned in dorsal recumbency is connected to the mechanical ventilator and ventilated with a mixture of air and oxygen (Respiratory Rate 16 breath/minutes, Tidal Volume 10 ml/Kg). Under aseptic conditions, a venous introducer is placed in the right jugular or in the femoral vein using a combination of the cutdown procedure and Seldinger’s technique to expose blood vessels and introduce the catheter [10,11].
3. Manteinance: it is performed by continuous intravenous infusion of propofol (6 mg/Kg/hour) or inhalathor administration of isoflorane (1,2%). plus butorphanol (0.5 mg/kg/hour iv) plus rocuronium bromide (0.8 mg/kg iv) plus ketamine in bolus during major surgical stress. Blood volume is maintained with the infusion of saline (hematocrit target 25–40%).
4. Recovery: After every experimental procedure, sedation is stopped and animals are positioned in sternal recumbency. After the reversion of palpebral and mastication reflexes and of spontaneus breath, animals are estubated and returned to their stall, where they are monitorized with water and food available, soft lighting, and an ambient temperature and humidity of 22+/−2% and 55+/−10% .
5. Euthanasia: after all the experiments animals are sacrificed by the intravenus injection with embutramide 200 mg plus mebenzonio iodide 50 mg plus tetracaine hydrochloride 5 mg (3 ml/10 kg). During the procedure pigs are sedated and connected to a mechanical ventilator.

With the introducer in place, a 5-French balloon tipped catheter (Swan-Ganz) is advanced through it under fluoroscopic guidance and hooked into a hepatic vein. Once the tip of the catheter is inside the main right hepatic vein the position of catheter and caliber of vein is confirmed by injection of contrast.

Free (FHVP) and wedged hepatic venous pressure (WHVP) are measured and recorded and the hepatic venous pressure gradient (HVPG) is calculated.

Just before these measurements, 5 ml of blood is drawn from a main hepatic vein and peripheral vein (PV) and analysed successively to measure plasma remifentanil concentration -or other drugs, administered by bolus or continuous infusion-, and allow metabolomics profiling.

Plasma samples are collected for determination of pharmacokinetic (including the hepatic extraction ratio) and pharmacodynamic (reflected in increases in remifentanilic acid levels, the main metabolite of remifentanil) characteristics of remifentanil.

The extraction ratio of remifentanil is determined as (peripheral vein plasma concentration–hepatic vein plasma concentration) / (peripheral vein plasma concentration).

Quantification of remifentanil in opportunely prepared plasma samples is performed by liquid chromatography coupled to tandem mass spectrometry (LC-MS/MS) [12].

Hepatic blood flow (HBF) will be measured using the indocyanine green (ICG) continuous infusion, if necessary.

## IV. DISCUSSION

Remifentanil has been selected as model drug to explore the concept of drug metabolism independent of liver function.

Remifentanil is a synthetic μ-opioid agonist; although chemically related to the fentanyl family of short-acting 4-an-ilidopoperidine derivatives, remifentanil is structurally unique among currently commercially available opioids secondary to an ester-linkage that is crucial to its opioid activity. Unlike other opioid analogs, it is susceptible to ester hydrolysis by blood and tissue esterases. This gives it an “ultra-short” duration of action and makes its elimination virtually independent on hepatic or renal function [13].

Its pharmacokinetic characteristics for what concerns hepatic metabolism have been explored in mainly during liver transplant [2].

Starting from this consideration we elaborated an experimental model, which we are going to submit to the animal studies ethical committee evaluation, that has the characteristic to be easier than an “anhepatic” phase of a transplant model and less invasive than a laparotomic cannulation of hepatic vein.

Obviously, many other drugs, which hepatic metabolism is currently under study, could be approached by this method and it could allow researchers to test liver metabolism of drug in different clinical settings. In critical care research, i.e., would be very interesting to study drugs pharmacokinetics in sepsis [14,15] or in severe hypotensive states [16], which are among the underlying conditions [17–19] to the administration of sedative drugs.

Nevertheless, we realize that our experimental model presents some difficulties, such as vein cannulation.

The external jugular veins of swine are located at the same tissue depth as the internal jugular veins and carotid sheath. The depth of their location makes identification of them difficult. Femoral access is generally gained by surgical incision: we propose a combination of incision and percutaneous approach, with Doppler guide.

Tunnelling of the catheter is auspicable for repeated measures: if the catheter is tunnelled, is it possible to repeat measures and withdrawals for many days or with different drugs, minimizing costs and avoiding sufferance to the experimental animals. Catheter access should be located on the dorsum of the body to prevent trauma but Swan Ganz catheter are not designed for tunnelisation.

This experimental model needs to be tested: in the mean while we are looking for other minimally invasive possibilities to study drug metabolism *in vivo*.

## Figures and Tables

**Table 1:** paper reporting the use of HVPG technique to collect blood from hepatic veins.

**Author, year**	**Procedure**	**Number of pts**	**Measurements of concentration**	**Results**
Tarquini R, 2012	HVPG	31 cirrhotic pts	CO, NO	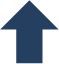 CO hepatic vein 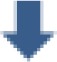 NO hepatic vein
Lebrec D, 2012	HVPG	24 cirrothic pts	Extraction ratio of tezosentan	0,28 (extraction on passage through the liver)
Avritscher R, 2001	HVPG and embolization	Experimental pigs	none	HVPG feasible in pigs

**Table 2:** experimental model to study liver extraction rate of drugs

**Author, year**	**Procedure**	**Experimental animal**	**Measurements of concentration**	**Results**
Kunta JR, 2001	Tunnelled catheter implanted in the portal vein	rabbit	none	Feasible technique
Mills PC, 2004	Catheters placed in the portal and hepatic veins during exploratory laparotomy	dog	propanolol	High hepatic extraction of propanolol
